# Mediating Role of Basic Psychological Needs in the Association Between Exercise Goal Content and Subjective Well-Being in Portuguese Older Adults

**DOI:** 10.3390/healthcare13172086

**Published:** 2025-08-22

**Authors:** Nuno Couto, Raul Antunes, Teresa Bento, Anabela Vitorino, Diogo Monteiro, Luís Cid

**Affiliations:** 1 Sport Sciences School of Rio Maior, Santarém Polytechnic University (ESDRM-IPSantarém), 2040-413 Rio Maior, Portugal; teresabento@esdrm.ipsantarem.pt (T.B.); anabelav@esdrm.ipsantarem.pt (A.V.);; 2 Research Centre in Sports Sciences, Health Sciences and Human Development (CIDESD), 5000-558 Vila Real, Portugal; raul.antunes@ipleiria.pt (R.A.); diogo.monteiro@ipleiria.pt (D.M.); 3 School of Education and Social Sciences (ESECS), Polytechnic University of Leiria (ESECS-IPLeiria), 2411-901 Leiria, Portugal

**Keywords:** motivation, satisfaction with life, positive affect, negative affect, basic psychological needs, older people

## Abstract

**Background**: This study aimed to examine the association between different exercise goals and their impact on subjective well-being (SWB) variables, namely, positive affect (PA), negative affect (NA), and satisfaction with life (SWL), as well as to explore the mediating role of basic psychological needs (BPNs) in this relationship within a sample of Portuguese older adults. **Methods**: The sample study constituted 298 individuals (233 females, 65 males), aged between 60 and 90 years (M = 68.43; SD = 6.48). Through model four of the Process macro for SPSS version 3.5, a simple mediation analysis was carried out. **Results**: The results show that BPNs mediated the relationship between goal content for exercise health management and PA and SWL; goal content for exercise skill development and PA; goal content for exercise image, PA, and SWL; and goal content for exercise social affiliation and PA. **Conclusions**: Thus, we can conclude that BPNs stand out as a relevant mediator in the relationship between goal content for exercise and SWB variables, which reinforces the importance of BPNs in SWB promotion in the older population.

## 1. Introduction

The most recent United Nations report projects that the global population aged 65 and over will double by 2050 [1]. Furthermore, the number of individuals aged 80 and above is expected to triple by 2050 compared to 2024 levels [2]. Population aging poses a significant challenge for societies worldwide, with anticipated demographic shifts likely to increase the burden on pension systems and healthcare services [3].

Aging is a continuous process marked by progressive changes in physical and mental health, largely driven by the accumulation of molecular and cellular damage over time [4]. In addition to biological changes, aging is often accompanied by life transitions—such as retirement or the loss of significant others, that can negatively affect the well-being of older adults [5,6]. Subjective well-being (SWB), defined as a long-term state characterized by the presence of positive affect (PA), satisfaction with life (SWL), and the absence of negative affect (NA) [7], is particularly sensitive to these factors in this population. Research has demonstrated a bidirectional relationship between physical health and SWB, as individuals with chronic conditions, such as arthritis, coronary heart disease, or chronic lung disease, tend to report lower levels of SWB [6,8]. It is therefore essential to acknowledge that the majority of health issues associated with aging are linked to chronic conditions, many of which can be prevented through the adoption of healthy behaviors across the lifespan [4]. In this context, active aging, which should be approached from a life course perspective [9], can be understood as the process of optimizing opportunities for health, participation, and security, with the ultimate goal of enhancing quality of life in later years [4]. Given the above, regular physical exercise plays a crucial role in older adults, as it positively impacts physical capacity [10] and SWB, notably by enhancing SWL [11] and PA [11,12] while also reducing NA [12]. Despite its recognized importance, epidemiological studies have shown a decline in regular physical activity with increasing age [13,14,15].

Therefore, understanding the reasons behind human involvement in a particular activity and how they persist with it is one of the central issues of social science research, and, in this sense, motivation is one of the topics that has been most investigated in various areas, as it is defined as a psychological variable that moves the individual towards performing, guiding, maintaining, or leaving a physical activity [16]. In this context, self-determination theory (SDT) (Deci & Ryan [17]) provides a comprehensive framework for explaining the quality of motivation and its consequences for well-being and behavioral regulation. A key component of SDT is the Goal Contents Theory, which distinguishes between intrinsic and extrinsic exercise goals. According to Sebire et al. (2008) [18], exercise goals can be grouped into five domains: image, health maintenance, social recognition, social affiliation, and skills development. Such goals comprise intrinsic reasons for engaging in physical activity as well as the variation in motivational quality. Intrinsic goals, such as health, social affiliation, skills development, personal growth, and self-fulfillment values, positively relate with BPN satisfaction, SWL, PA, and exercise persistence. Extrinsic goals, such as image and social recognition, are associated with external demands as well as social approval and tend to align with lower BPN satisfaction, lower SWB (i.e., lower SWL and PA, escalating NA), and exercise dropout.

Another core aspect of SDT is that self-actualization is a central aspect of well-being and elucidates the extent to which the satisfaction of the basic psychological needs (BPNs) of autonomy (i.e., the ability to regulate one’s own actions), competence (i.e., the subject’s ability to be effective in interacting with involvement), and relatedness (i.e., the ability to seek out and develop interpersonal connections and relationships) [19], three “fundamental nutrients” [20], are important for psychological growth, integrity (e.g., internalization and assimilation of cultural practices), and well-being (e.g., psychological health). Satisfaction of BPNs is critical for the development of autonomous behavioral regulations, for example, intrinsic motivation, defined as performing an activity for the inherent pleasure and enjoyment it brings (e.g., exercising because one enjoys it or finds it personally meaningful). Such a regulation is linked to greater well-being and long-term behavioral compliance over time [21,22]. In comparison, extrinsic motivation represents behaviors motivated by external rewards or pressures (e.g., exercising to gain praise or fear criticism), according to SDT. On the other hand, the thwarting of BPNs results in behavioral regulation shifting towards being more controlled (e.g., external regulation), which is negatively linked with well-being and long-term compliance with behavior [23,24].

Evidence from previous studies indicates that intrinsic goals for exercise (i.e., health and skills development) are positively associated with satisfaction of BPNs; SWL and PA [25,26,27,28,29]; and with more frequency and persistence of exercise behavior [30]. On the other hand, extrinsic goals (i.e., social affiliation, image, and social recognition) are negatively associated with BPN satisfaction, whereas SWL and PA [26,27,28,30] are positively related to NA [27]. Regarding exercise, those who regulate their behavior through extrinsic goals are more linked to reduced frequency and dropout [27,31].

Despite the influence of the content of exercise goals on BPNs, SWB, and exercise adherence, there has been little research into these variables in the older population. Apart from the study by Rodrigues et al. [25], which grouped intrinsic and extrinsic goals into a composite factor and analyzed how they influenced only the cognitive variable of SWB (i.e., SWL) and exercise adherence through the influence of BPNs in a sample of older people, most of the studies identified above use samples made up of people under 65 years old. Previously, Antunes et al. [32] studied the effect of different exercise contents on SWB variables (i.e., SWL, PA, NA) in older people. However, in this study, the role of BPNs in this sequence was not assessed.

Although numerous studies have examined this topic in younger populations (i.e., under 65 years) [27], there remains a critical need to focus on older adults due to the established importance of physical activity and subjective well-being (SWB) in this age group [10,11,12] as well as the influential role of basic psychological needs (BPNs) in regulating behavior and SWB [22,26]. Therefore, the present study aims to investigate the association between different exercise goal contents—both extrinsic and intrinsic—and SWB variables (positive affect, negative affect, and satisfaction with life) in older adults while also exploring the mediating role of BPNs in this relationship.

## 2. Materials and Methods

### 2.1. Participants and Procedures

A total of 299 Portuguese older people (234 female, 65 male) enrolled in senior universities and day centers (none of them were institutionalized) living in Ribatejo and western Portugal, with ages between 60 and 90 years (M = 68.43, SD = 6.48), participated in the study. Of the total sample, 79.7% reported engaging in regular physical activity with a frequency ranging from 1 to 7 times per week (M = 1.73; SD = 1.53). Among the activities reported, the most common were maintenance exercise, aerobics, water aerobics, and walking.

After contacting the administrations of the senior universities and nursing homes to obtain signatures on the informed consent forms from the participants, all the data were anonymously collected and analyzed, thereby assuring compliance with the confidentiality principle. To provide further details, the data were collected in a classroom context in the local facilities of the senior universities in small groups (maximum of 20 people), with a duration of approximately 20 min. This study was approved by the ethics committee of the Regional Health Administration of Lisbon and Tagus Valley (ARSLVT), having received a favorable opinion for its implementation: Opinion 129/CES/INV/2013 from the Ethics Committee on 1 April 2014.

### 2.2. Instruments

The Goal Content for Exercise Questionnaire (GCEQ) developed by Sebire et al. [18], validated for Portuguese older people [32], allows the assessment of the content of goals for physical exercise practice. It consists of 20 items, which are answered on a Likert scale with seven response levels, ranging from one (“strongly disagree”) to seven (“strongly agree”). Subsequently, the items are grouped into factors, each one with four items. This questionnaire allows assessment of three intrinsic factors (health maintenance—e.g., “To improve my overall health”; skill development—e.g., “To learn and practice new exercises and/or activities”; social affiliation—e.g., “To share my exercise practice experiences with people who care about me

(“and two extrinsic factors (image—e.g., “To improve the overall image of my body”; social recognition—e.g., “So that others have a good impression of me”).

The Basic Need Satisfaction General Scale (BNSG-S) developed by Gagné [33], validated for Portuguese older people [34], allows for the assessment of the overall satisfaction of BNS in the lives of Portuguese seniors. This instrument consists of 13 items, to which responses are given on a Likert scale with seven response levels. The items are subsequently grouped into three factors (i.e., autonomy, three items; competence, three items; relatedness, five items) that reflect the basic psychological needs underlying self-determination theory (SDT: Deci & Ryan [17]).

The Positive and Negative Affect Schedule (PANAS) developed by Watson et al. [35], validated for Portuguese older people [12], comprises 20 items, which are answered using a Likert-type scale with five levels that vary between 1 (“nothing or very slightly”) and 5 (“extremely”). Then, the items are grouped into two factors that represent the degree of positive (e.g., “interested”, “strong”, “excited”) and negative (e.g., “perturbed”, “scared”, “angry”) affect.

The Satisfaction With Life Scale (SWLS) developed by Diener et al. [36], validated for Portuguese older people [37], comprises five items, which are answered using a Likert-type scale with seven levels that vary between one (“totally disagree”) and seven (“totally agree”). Afterwards, the items are grouped into only one factor, which presents an index of overall satisfaction with life.

### 2.3. Statistical Analysis

Through IBM SPSS software version 27.0 (IBM Corp., Armonk, NY, USA, 2020), descriptive statistics and correlation analysis between the study variables were conducted. The PROCESS macro for SPSS version 3.5 was also used for simple mediation analysis, which, according to Hayes [38], demonstrates how the effect of a variable on an outcome, quantified through ordinary least squares (OLS) regression, can be divided into direct and indirect effects. Model four (model as a parameter in the PROCESS function) was used, which, according to Hayes [38], allows for the evaluation of the direct effect of the independent variable on the dependent variable and the indirect effect of the two variables through the mediating variable.

To calculate the statistical significance of a deviation from the normal distribution, skewness and kurtosis were estimated. According to Kline [39], normality is assumed when the absolute values of skewness are less than 3 and kurtosis less than 8.

The Bootstrap method with 5000 samples and a 95% confidence level was also used to calculate the direct and indirect effects of the contents of intrinsic and extrinsic motives on subjective vitality. If 95% of the confidence interval is negative or positive, the mediation model is considered significant. Fritz and Mackinnon [40] indicate that the current sample size aligns with simulation studies for mediation analysis involving this number of variables, thereby ensuring adequate statistical power.

Before carrying out the main analyses, the possible impact of common method bias (CMB) was evaluated since all the variables were measured through self-report questionnaires administered at a single time and filled in by the very same participants. Such a design heightens the threat of spuriously shared variance among constructs [41]. To correct this, Harman’s single-factor test was administered. All scale measurement items from the measures administered in the study (i.e., GCEQ, BPNG-S, PANAS, and SWL) were included in an unrotated principal component analysis to test the proportion of variance explained in the first extracted factor. More than a 40% variance explanation is conventionally identified as significant CMB [42].

## 3. Results

The results showed that the variance explained by the first unrotated factor was 16.22, below the 40% threshold. These findings indicate that CMB in this study is minimal and acceptable, suggesting that the associations among variables are unlikely to be influenced by shared measurement methods. The univariate normality was supported, as skewness and kurtosis values fell within the acceptable thresholds of ±3 and ±8, respectively.

Descriptive analysis was carried out as well as a correlations analysis between the study’s variables. Table 1 shows that the health management goal was the most valued, while social recognition was the least valued. Regarding SWB variables, SWL was the most valued, while NA was the least valued. In terms of the correlations between the variables under analysis, it was found that all the objectives are significantly related (i.e., *p* < 0.05) to BPNs. Regarding correlations between goal content for exercise and SWB variables, there was a positive and significant relationship between PA and health management, skill development, image, and social affiliation goals; NA and image, social recognition, and social affiliation; and between SWL and health management and image.

**Figure 1:**
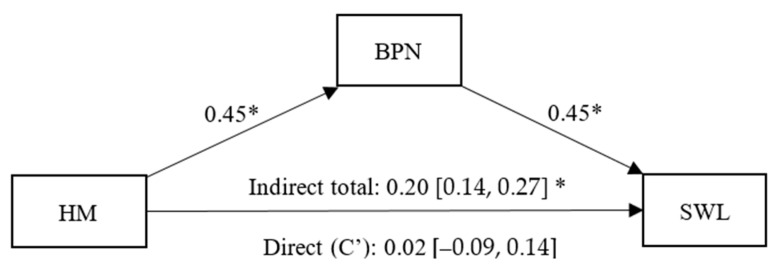
Simple mediation model between Health Management (HM), Basic Psychological Needs (BPN) and Satisfaction With Life (SWL). Note: * *p* < 0.05.

As shown in Figure 1, there is a positive and significant effect of health management on BPNs (β = 0.45, *p* < 0.01, CI 95% [0.35, 0.55]) and of BPNs on SWL (β = 0.45, *p* < 0.01, IC95% [0.33, 0.56]). A non-significant direct effect (C’) of health management on SWL was also found (β = 0.02, *p* = 0.02, CI 95% [−0.09, 0.14]). The indirect effect of health management on SWL, fully mediated by BPNs, was statistically significant (β = 0.20, CI 95% [0.14, 0.27]).

**Figure 2:**
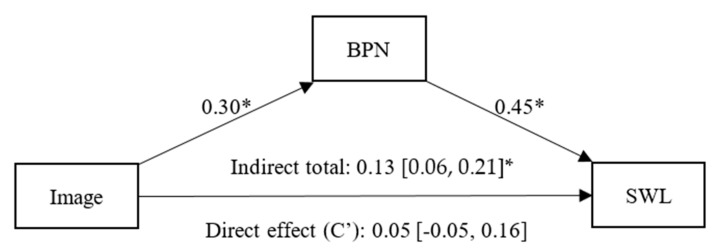
Simple mediation model between Image, Basic Psychological Needs (BPN) and Satisfaction With Life (SWL). Note: * *p* < 0.05.

Regarding Figure 2, in the mediation model, we identified a positive and significant effect of image on BPNs (β = 0.30, *p* < 0.01, CI 95% [0.19, 0.40]), and BPN satisfaction had a statistically significant effect on SWL (β = 0.45, *p* < 0.01, CI 95% [0.33, 0.55]). There was a non-significant direct effect (C’) of image on SWL (β = 0.05, *p* = 0.34, CI 95% [−0.05, 0.16]), and a significant indirect effect of image on SWL, fully mediated by BPNs (β = 0.13, CI 95% [0.06, 0.21]).

**Figure 3:**
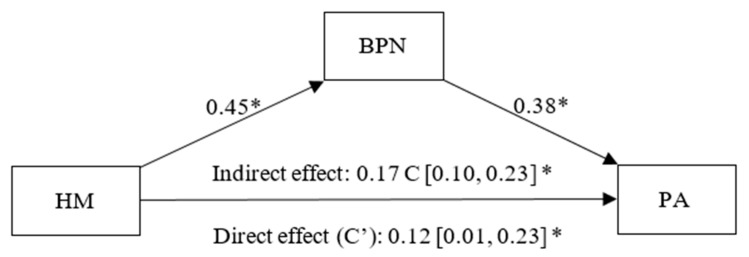
Simple mediation model between Health Management (HM), Basic Psychological Needs (BPN) and Positive Affect (PA). Note: * *p* < 0.05.

In Figure 3, we can observe the presence of a positive and significant effect of health management on BPNs (β = 0.45, *p* < 0.01, CI 95% [0.35, 0.55]) and of BPNs on PA (β = 0.38, *p* < 0.01, CI 95% [0.26, 0.49]). A significant direct effect (C’) of health management on PA was also found (β = 0.12, *p* = 0.04, CI 95% [0.01, 0.23]). Additionally, a statistically significant indirect effect of this goal on PA, partially mediated by BPNs, (β = 0.17, CI 95% [0.10, 0.23]) was observed.

**Figure 4:**
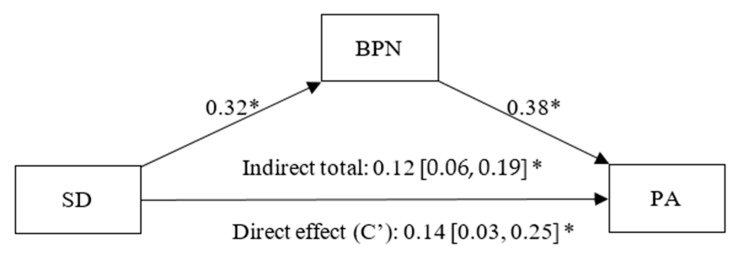
Simple mediation model between Skill Development (SD), Basic Psychological Needs (BPN) and Positive Affect (PA). Note: * *p* < 0.05.

For skill development, as shown in Figure 4, the simple mediation model evidences a positive and significant effect of this goal on BPNs (β = 0.32, *p* < 0.01, CI 95% [0.20, 0.42]) and of BPNs on PA (β = 0.38, *p* < 0.01, CI 95% [0.28, 0.50]). A significant direct effect (C’) of skill development on PA (β = 0.14, *p* = 0.09, CI 95% [0.03, 0.25]) was also found. A statistically significant indirect effect of skill development on PA, partially mediated by BPNs, was also observed (β = 0.12, CI 95% [0.06, 0.19]).

**Figure 5:**
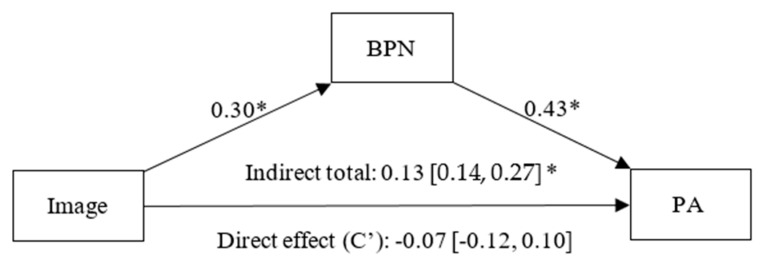
Simple mediation model between Image, Basic Psychological Needs (BPN) and Positive Affect (PA). Note: * *p* < 0.05.

As observed in Figure 5, the goal content of image has a positive and significant effect on BPNs (β = 0.30, *p* < 0.01, CI 95% [0.19, 0.40]), and BPNs have a significant positive effect on PA (β = 0.43, *p* < 0.01, CI 95% [0.32, 0.54]). A non-significant direct effect (C’) of image on PA was found (β = −0.07, *p* = 0.90, CI 95% [−0.12, 0.10]), while a statistically significant indirect effect of image on PA, fully mediated by BPNs (β = 0.13, CI 95% [0.05, 0.20]), was also observed.

**Figure 6:**
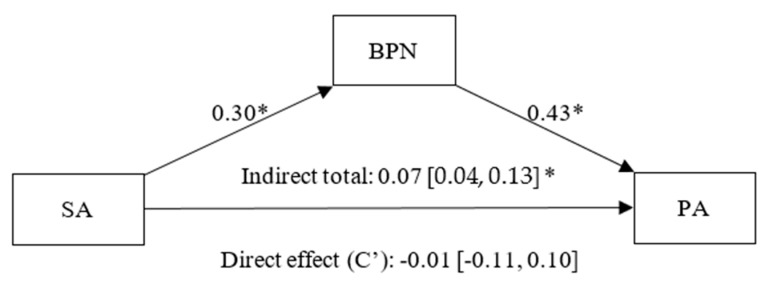
Simple mediation model between Social Affiliation (SA), Basic Psychological Needs (BPN) and Positive Affect (PA). Note: * *p* < 0.05.

As shown in Figure 6, the goal content of social affiliation has a positive and significant effect on BPNs (β = 0.30, *p* < 0.01, CI 95% [0.19, 0.40]), and BPNs have a statistically significant effect on PA (β = 0.43, *p* < 0.01, CI 95% [0.32, 0.54]). A statistically non-significant direct effect (C’) of social affiliation on PA was found (β = −0.01, *p* = 0.89, CI 95% [−0.11, 0.10]), while a significant indirect effect of social affiliation on PA, fully mediated by BPNs (β = 0.07, CI 95% [0.04, 0.13]) was also observed.

**Figure 7:**
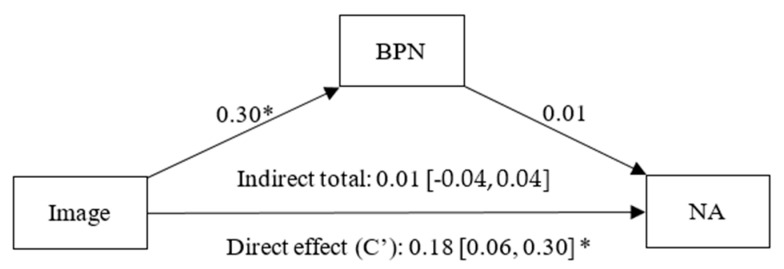
Simple mediation model between Image, Basic Psychological Needs (BPN) and Negative Affect (NA). Note: * *p* < 0.05.

Through the mediation model related to NA, it was identified that the goal content of image (Figure 7) had a positive effect on BPNs (β = 0.30, *p* < 0.01, CI 95% [0.19, 0.40]), and a non-significant effect of BPNs on NA was identified (β = 0.01, *p* = 0.98, CI 95% [−0.11, 0.11]). A significant direct effect (c’) of image on NA was also observed (β = 0.18, *p* < 0.01, CI 95% [0.06, 0.30]), whereas the indirect effect of image on NA through BPNs was statistically non-significant (β = 0.01, CI 95% [−0.04, 0.04]).

**Figure 8:**
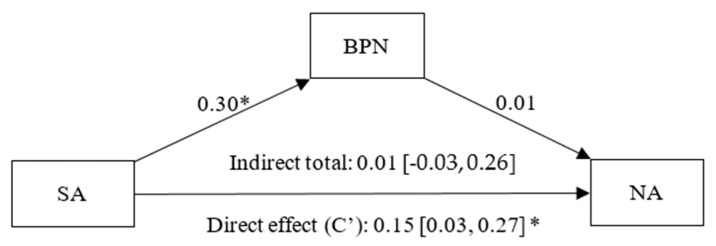
Simple mediation model between Social Affiliation (SA), Basic Psychological Needs (BPN) and Negative Affect (NA). Note: * *p* < 0.05.

Through Figure 8, it was identified that social affiliation had a positive and significant effect on BPNs (β = 0.30, *p* < 0.01, CI 95% [0.19, 0.40]), BPN satisfaction had a statistically non-significant effect on NA (β = 0.01, *p* < 0.88 CI 95% [−0.11, 0.13]), and there was a statistically significant direct effect (C’) of SA on NA (β = 0.15, *p* < 0.01, CI 95% [0.03, 0.27]) and a non-significant indirect effect of SA on NA, not mediated by BPNs (β = 0.01, CI 95% [−0.03, 0.26]).

**Figure 9:**
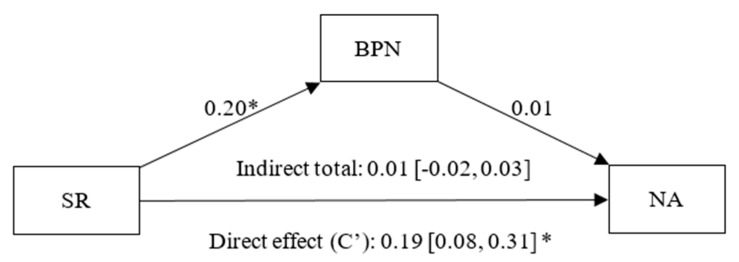
Simple mediation model between Social Recognition (SR), Basic Psychological Needs (BPN) and Negative Affect (NA). Note: * *p* < 0.05.

Finally, in Figure 9, it was identified that social recognition has a positive and significant effect on BPNs (β = 0.20, *p* < 0.01, CI 95% [0.09, 0.31]), BPN satisfaction has a statistically non-significant effect on NA (β = 0.01, *p* = 0.82, CI 95% [−0.10, 0.13]), and there is a statistically significant direct effect (C’) of SR on NA (β = 0.19, *p* < 0.01, CI 95% [0.08, 0.31]) and a non-significant indirect effect of SR on NA, not mediated by BPNs (β = 0.01, CI 95% [−0.02, 0.03]).

## 4. Discussion

This study aimed to examine the association between different exercise goals and their impact on subjective well-being (SWB) variables—namely, positive affect (PA), negative affect (NA), and satisfaction with life (SWL)—as well as to explore the mediating role of basic psychological needs (BPNs) in this relationship within a sample of Portuguese older adults. Prior to conducting the mediation analysis, descriptive results revealed that health management is the most highly valued goal among older adults for engaging in physical exercise, whereas social recognition is the least valued. This finding is consistent with previous research, which highlights that older individuals primarily view exercise as a means to enhancing health and maintaining independence—goals that are particularly salient in this life stage [43]. This pattern of goal prioritization aligns with the Socioemotional Selectivity Theory [44], which posits that as individuals age, they increasingly prioritize emotionally meaningful goals and immediate well-being over social status or long-term aspirations.

Regarding the SWB variables, participants in the present sample reported moderate to high levels of SWL and PA, along with relatively low levels of NA. These results suggest that the participants experience fewer negative emotions and maintain a generally positive perception of their life and emotional state. Previous research supports this finding, indicating that perception of SWB is not necessarily dependent on age. Individuals at different life stages may report higher levels of PA and SWL, and lower levels of NA, when they frequently experience positive emotions and evaluate their lives positively from a cognitive standpoint [45]. Although contextual and cultural differences may influence outcomes, studies consistently show that older adults can maintain high levels of SWL and PA, particularly when they benefit from reasonable physical health, strong social support, and positive psychological adjustment [6,44].

In terms of the correlation analysis, several significant associations were found between intrinsic and extrinsic exercise goals and the SWB variables.

The intrinsic goal of health management was found to be significantly correlated with both PA and SWL, suggesting that older adults who engage in exercise for health-related reasons tend to report more positive emotions and a greater cognitive appraisal of SWL. As noted by Deci and Ryan [26], health management motivation involves proactive behaviors, such as regular exercise and healthy eating, which enhance autonomy, foster a sense of control over one’s life, and promote self-efficacy. These psychological resources are associated with increased PA and a more positive evaluation of one’s life circumstances. Additionally, the intrinsic goal of skill development was positively and significantly correlated with PA but not with SWL. Skill development refers to the ongoing process of acquiring and refining cognitive, interpersonal, and intrapersonal competencies that enable individuals to perform complex tasks, solve problems, and adapt effectively to new challenges [46]. In the exercise domain, Sebire et al. [28] conceptualized skill development as an intrinsic goal that reflects the motivation to improve and acquire new physical and technical abilities. While this goal is strongly associated with emotional well-being, particularly the experience of positive emotions such as enjoyment, competence, and satisfaction, it appears to have a limited effect on cognitive evaluations of life, such as SWL [47]. This distinction may be explained by the fact that SWL is a cognitive judgment shaped by the pursuit and achievement of enduring, meaningful goals [48,49] and is less influenced by transient affective states or short-term emotional experiences [36].

With respect to extrinsic goals for exercise (i.e., image, social affiliation, social recognition), distinct correlations were verified between them and SWB variables. The exercise goal of image had a positive and significant correlation with PA, NA, and SWL. Considering the extrinsic nature of this goal, it was not expected that it could be positively significantly correlated with PA and SWL. According to Antunes et al. [32], their study validating the GCEQ in Portuguese older people also found a positive relationship between health management and image. The authors considered that these individuals understood the reasons for their exercise practice in terms of image as health promotion (i.e., image can be a factor in a healthy state). Research has found that when older people exercise to improve their image, self-care, self-esteem, and social identity [50], it can improve their physical condition and ability to experience more positive emotions [31,50]. In addition, the perception that older people are still able to take care of themselves and their appearance and to excel socially may increase feelings of control and personal fulfillment, both of which are important predictors of SWL [51,52]. Nevertheless, this goal can negatively affect older people’s well-being through frustration if it is associated with social pressure and unattainable beauty ideals [53,54,55].

Finally, although both affiliation and recognition are social goals, they have a different response to SWB. The goal of social recognition, being an extrinsic value aimed at approval and status in the eyes of others, tends to correlate positively only with NA, such as anxiety, frustration, and insecurity, especially when individuals perceive that they are not being valued or recognized as they expect [26,53]. The constant search for external validation can make emotional well-being unstable, generating emotional distress even in the face of apparent achievements [56]. On the other hand, the goal of social affiliation, aimed at building meaningful bonds and belonging, tends to correlate positively with both PA and NA since interpersonal relationships are sources of intense and ambivalent emotions. When there is connection and support, PA predominates. However, in situations of conflict, rejection, or loneliness, NA emerges [57,58,59]. Thus, the nature of social goals and the relational context in which they manifest themselves directly influence the type of affect experienced by individuals.

Regarding BPNs, positive correlations were observed between all exercise goals and overall BPN satisfaction. According to the theoretical framework, intrinsic goals are typically expected to be positively associated with BPN satisfaction [60,61]. Interestingly, in this study, the extrinsic goals of social affiliation, social recognition, and image were also positively correlated with BPNs. This pattern can be partly explained by the nature of these goals. Although categorized as extrinsic, the goal of social affiliation is inherently rooted in the development of interpersonal relationships and is closely aligned with the need for relatedness, one of the core components of BPNs [53]. Similarly, social recognition, while extrinsic, can be linked to the satisfaction of the need for competence. When individuals engage in behaviors that are socially acknowledged, such recognition may reinforce their perception of competence, thereby supporting psychological need satisfaction [62]. In fact, previous research has shown that authentic praise for individual effort and initiative can enhance the satisfaction of the competence need [26,63]. Finally, the positive correlation between the image goal and BPN satisfaction may be interpreted through the lens of recent findings by Antunes et al. [32], who observed a positive association between image-related motives and health, an intrinsic goal. These authors suggested that in older adults, image is not necessarily perceived as a superficial or extrinsic goal but rather as a representation of physical fitness and a marker of a healthy lifestyle. In this sense, motives related to image may be internalized and reinterpreted as indicators of health promotion, contributing indirectly to the satisfaction of BPNs.

Regarding BPNs, the results revealed positive and significant correlations with SWL and PA and a non-significant correlation with NA. These findings are consistent with SDT, which emphasizes the crucial role of the need for relatedness, competence, and autonomy in promoting well-being. When these psychological needs are consistently satisfied throughout life, older adults are more likely to develop greater emotional resilience, experience higher levels of PA, and maintain a more positive cognitive evaluation of their lives, all of which are positively associated with subjective well-being [26].

Considering the presence of various correlations between variables, mediation analyses were developed between them since assessing the mediation of another factor between variables that are not related is unusual [38]. In this way, it was observed that BPNs totally mediate the relationship between health management and SWL, image and PA, image and SWL, and social affiliation and PA. These results suggest that these goals have a positive impact on well-being when they effectively promote the satisfaction of autonomy, competence, and relationship needs. According to SDT, it is the satisfaction of these psychological needs that sustains optimal functioning and SWB, and the goals themselves are not sufficient to generate such effects [20,26]. On the other hand, the partial mediation observed in the relationship between health management, skill development goals, and PA indicates that, although part of the effect goes through the satisfaction of BPNs, there are also direct effects of these goals on SWB variables. This may be because these goals have a more self-determined and intrinsically motivating content, promoting PA both by supporting BPNs and, independently, by reinforcing the perception of self-care and personal growth [64]. Thus, the data reinforce the centrality of BPNs as fundamental mechanisms in the relationship between motivation to exercise and well-being, highlighting the mediating role of these needs in the context of exercise in older people.

Regarding the limitations of this study, it is important to acknowledge certain factors that may influence the interpretation and generalization of the findings. Highlighting these limitations does not undermine the value of the results; rather, it encourages a critical and informed understanding of the research. Firstly, the cross-sectional design restricts the ability to establish causal relationships between the study variables, allowing only for the observation of associations at a single point in time. Secondly, the study was conducted with a relatively small and specific segment of the population, which, although justified, may limit the generalizability of the findings to the broader Portuguese older adult population. Despite these constraints, the study offers valuable insights into the influence of exercise goal content and BPNs on SWB in later life. Furthermore, the findings highlight meaningful directions for future research, particularly using complementary methodologies that can deepen understanding of these relationships over time.

One limitation of the present study concerns the exclusive use of self-report instruments, which may introduce biases such as social desirability or errors in subjective perception. The inclusion of objective indicators, such as behavioral assessments, observational data, or physiological measures, could have contributed to a more robust methodological triangulation and a broader understanding of the constructs analyzed. Future studies may benefit from adopting multimethod approaches to deepen the analysis of the phenomena investigated.

Another limitation in the current study is the lack of direct measures on chronic conditions or subjective health status. Such variables might indirectly affect motivation and subjective well-being, especially in older adults. Nevertheless, the sample included only functionally independent older adults, suggesting a minimum level of physical and cognitive well-being. Nevertheless, future research needs to account for the direct measurement of these variables to more adequately examine their role as potential moderators or covariates in the relationships between motivational regulation, BPN satisfaction, and emotional outcomes.

Future studies may investigate possible differences in the relationships between goals, SWB, and BPNs based on sociodemographic variables such as gender, even within the elderly population, which could contribute to a deeper understanding of these phenomena.

## 5. Conclusions

The aim of this work was to analyze the relationship between different exercise goals and their effect on SWB variables (i.e., PA, NA, and SWL) and to understand the mediating role of BPNs between these two constructs in a sample of Portuguese older adults. Regarding correlational analysis, we can conclude that there are positive and significant relationships between goal content for exercise health management, skill development, social affiliation, social recognition, and BPNs; between goal content image, social recognition, social affiliation, and NA; and finally, between goal content health management, image, and SWL. According to the mediation analysis, we can conclude that BPN satisfaction mediates the relationship between health management goal content and PA and SWL; exercise skill development goal content and PA; exercise image goal content, PA, and SWL; and exercise social affiliation goal content and PA. Thus, we can conclude that BPNs stand out as a relevant mediator in the relationship between exercise goal content for exercise and SWB variables, which reinforces the importance of BPNs in promoting SWB in the older population.

Thus, this study reveals that exercise motives for older adults can have different influences on PA and NA, as well as on SWL, and that they may be mediated by BPN satisfaction. In that respect, it certainly appears that more self-determined reasons for exercise, such as being active for mobility preservation, for everyday independence, or for relationships, make for more success in this age demographic.

Therefore, all fitness specialists who work with older adults need to scrutinize and promote strong personal goals for physical activity; develop group-centered programs that enhance a sense of membership; engage in activities that are adjusted according to personal limitations; and provide day-after-day positive reinforcement that values effort. Exercise specialists are further recommended to try to make exercise aims compatible with a focus on the psychological fundamentals of autonomy, competence, and relatedness. Towards that end, attempts like permitting choice between exercises (improving autonomy), incorporating exercises that enhance a sense of functional ability/skill development (enhancing competence), and forming positive social interactions/affective relationships between members of a session (enhancing relatedness) could be made.

Such approaches are consistent with the theoretical framework adopted in this study and may contribute to the SWB of the older population.

## Figures and Tables

**Table 1:** Descriptive statistics and correlational analysis.

	1	2	3	4	5	6	7	8	9
1—Health Management	-								
2—Skill Development	0.48 **	-							
3—Image	0.45 **	0.56 **	-						
4—Social Recognition	0.45 **	0.52 **	0.56 **	-					
5—Social Affiliation	0.39 **	0.67 **	0.52 **	0.64 **	-				
6—BPNs	0.45 **	0.32 **	0.30 **	0.21 **	0.30 **	-			
7—PA	0.28 **	0.26 **	0.12 *	−0.02	0.12 *	0.43 **	-		
8—NA	0.57	0.10	0.17 **	0.20 **	0.15 **	0.53	0.41	-	
9—SWL	0.22 **	0.96	0.18 **	0.71	0.33	0.46 **	0.28 **	−0.27 **	-
Mean	6.01	5.06	4.93	3.80	4.88	5.42	3.28	1.93	4.71
Standard Deviation	0.79	1.14	1.10	1.38	1.14	0.67	0.69	0.71	1.00
Skewness	−0.84	−0.80	−0.76	−0.18	−0.86	−0.90	−1.41	0.98	−0.25
Kurtosis	1.14	1.60	1.77	−0.31	1.54	0.86	0.07	1.08	0.37

Notes: BPNs = Basic Psychological Needs; PA = Positive Affect; NA = Negative Affect; SWL = Satisfaction with Life; * *p* < 0.05; ** *p* < 0.01.

## Data Availability

Data are available upon request to the first author.
